# DIVIS: Integrated and Customizable Pipeline for Cancer Genome Sequencing Analysis and Interpretation

**DOI:** 10.3389/fonc.2021.672597

**Published:** 2021-06-08

**Authors:** Xiaoyu He, Yu Zhang, Danyang Yuan, Xinyin Han, Jiayin He, Xiaohong Duan, Siyao Liu, Xintong Wang, Beifang Niu

**Affiliations:** ^1^ Computer Network Information Center, Chinese Academy of Sciences, Beijing, China; ^2^ University of Chinese Academy of Sciences, Beijing, China; ^3^ ChosenMed Technology (Beijing) Co., Ltd., Beijing, China

**Keywords:** variants detection, customization, workflow, next-generation sequencing, cancer

## Abstract

Next-generation sequencing (NGS) has drastically enhanced human cancer research, but diverse sequencing strategies, complicated open-source software, and the identification of massive numbers of mutations have limited the clinical application of NGS. Here, we first presented GPyFlow, a lightweight tool that flexibly customizes, executes, and shares workflows. We then introduced DIVIS, a customizable pipeline based on GPyFlow that integrates read preprocessing, alignment, variant detection, and annotation of whole-genome sequencing, whole-exome sequencing, and gene-panel sequencing. By default, DIVIS screens variants from multiple callers and generates a standard variant-detection format list containing caller evidence for each sample, which is compatible with advanced analyses. Lastly, DIVIS generates a statistical report, including command lines, parameters, quality-control indicators, and mutation summary. DIVIS substantially facilitates complex cancer genome sequencing analyses by means of a single powerful and easy-to-use command. The DIVIS code is freely available at https://github.com/niu-lab/DIVIS, and the docker image can be downloaded from https://hub.docker.com/repository/docker/sunshinerain/divis.

## Introduction

Deciphering human cancer genome sequencing data is critical for the mapping of tumorigenesis and the development of targeting therapeutic strategies. The major focus of this research field is on cancer driver genes (CDGs) and cancer susceptibility genes (CSGs): CDGs are genes in which mutations confer cells a growth advantage that helps tumors proliferate (1), and CSGs are genes in which mutations, typically inherited, increase the risk of certain types of cancer (2). Studies on CDGs and CSGs are mainly based on the detection of somatic mutations and germline variants, respectively (2, 3). Although diverse methods for detecting and characterizing tumor variants have been published, it is challenging to identify all variants by using a single strategy (4). Moreover, independent algorithms are neither interoperable nor integrated because of the varying output from distinct algorithms. Furthermore, poor consistency between algorithms and complex configuration and parameters places a substantial burden on cancer genome analysis.

Various bioinformatics pipelines or platforms for cancer genome sequencing were developed over the past decade to overcome the limitations mentioned earlier. HugeSeq (5) combined the results of SAMtools (6) and GATK (7) to obtain short mutations, and identify structural variants (SVs) and copy number variants (CNVs) by using BreakDancer (8, 9), CNVnator (10), Pindel (11), and Break-Seq (12). However, the HugeSeq pipeline depended on a highly specialized computing environment and has not been updated and maintained for at least five years. Cake (13), a pipeline based on Perl scripts, integrated single nucleotide variants (SNVs) from four callers, Bambino (14), CaVEMan (15), SAMtools (6), and VarScan 2 (16), and accepted input in the BAM format, which avoided the complicated conversion from FASTQ to the BAM format. Another genome analysis pipeline, Fastq2vcf (17), concurrently detected SNVs and indels (insertions and deletions) and reported the consensus variants obtained from four different callers: GATK UnifiedGenotyper, GATK HaplotypeCaller, SAMtools, and SNVer (18). SeqMule integrated multiple-alignment and variant-calling tools to meet distinct combination requirements and supported deployment in computer clusters and the cloud (19), although it still requires external software to be installed and configured in advance. The cloud-based platform GenomeVIP (Genome Variant Investigation Platform) enabled the identification of somatic mutations and germline variants in whole-genome sequencing (WGS) and whole-exome sequencing (WES) (20), but the uploading and the security management of large sequencing data are major bottlenecks with this platform. iWhale (21), a customizable pipeline based on SCons (22), allows the inclusion of any bioinformatics tool, although software expansion or update still requires massive repetitive command lines and intricate programming.

The functional features of the software are listed in **Table 1**. Most of this software use Linux scripting or other programming languages (such as Python, Perl and R) to build the execution procedures in advance to realize the automatic execution of the whole process. Another drawback of using scripts is the difficulty of customization. When some software or parameters in the script need to be updated or replaced, it will involve the editing of integrated scripts, the reconstruction of pre and post logic, etc., which is not only easy to cause manual operation errors, but also cannot be generally applied to multi-feature genomic data analysis.

**Table 1:** Pipeline software of somatic mutations calling.

Software	Based-on	Input	Output	Sequencing	Mutations	Docker Image	Parallel
IWhale (21)	SCons	FASTQ	VCF (annotated)	WES	SNVs, indels	√	√
GenomeVIP (20)	Perl Scripts	BAM	VCF (annotated)	WGS, WES	SNVs, indels,SVs	×	×
SeqMule (19)	Bash Scripts	FASTQ	TXT (annotated)	WGS, WES	SNVs, indels	×	√
Fastq2vcf (17)	Bash Scripts	FASTQ	VCF (annotated)	WES	SNVs, indels	×	√
Cake (13)	Bash Scripts	BAM	VCF	WGS, WES, Panel	SNVs	×	√
HugeSeq (5)	Bash Scripts	FASTQ	VCF, GFF	WGS, WES	SNVs, indels	×	√
DIVIS	GPyFlow	FASTQ, BAM	VCF, MAF, HTML	WGS, WES, Panel	SNVs, indels	√	√

Bioinformatics development has promoted the continuous updating of software as well as the emergence of new bioinformatics tools; this requires high scalability and customizability of the analysis pipelines. Workflow management software such as Snakemake (23), Bpipe (24), Ruffus (25), Nextflow (26), and Galaxy (27) sufficiently satisfy this requirement by automatically generating analysis scripts through graphical operation, which simplifies human intervention. Snakemake, inspired by GNU Make, describes a workflow by using its Domain Specific Language (DSL) and executes the workflow through Python scripts (23). Similarly, Bpipe also describes a workflow by using its DSL, which is implemented in Groovy and run on Java Virtual Machine (24). Ruffus is a lightweight Python library that provides “split”, “transform”, “merge”, “collate”, and other operators to create a workflow (25). Nextflow also describes a workflow by using its DSL and generates scalable and reproducible scientific workflows by using software containers (26). Galaxy, a web-based platform, provides users with not only a visual workflow editor, but also several bioinformatics tools (27). Although these software tools perform effectively in workflow execution and management, most of them require user knowledge of DSL or specific programming language Common Workflow Language). Moreover, some of the software lack an interactive graphical interface.

**Figure 1:**
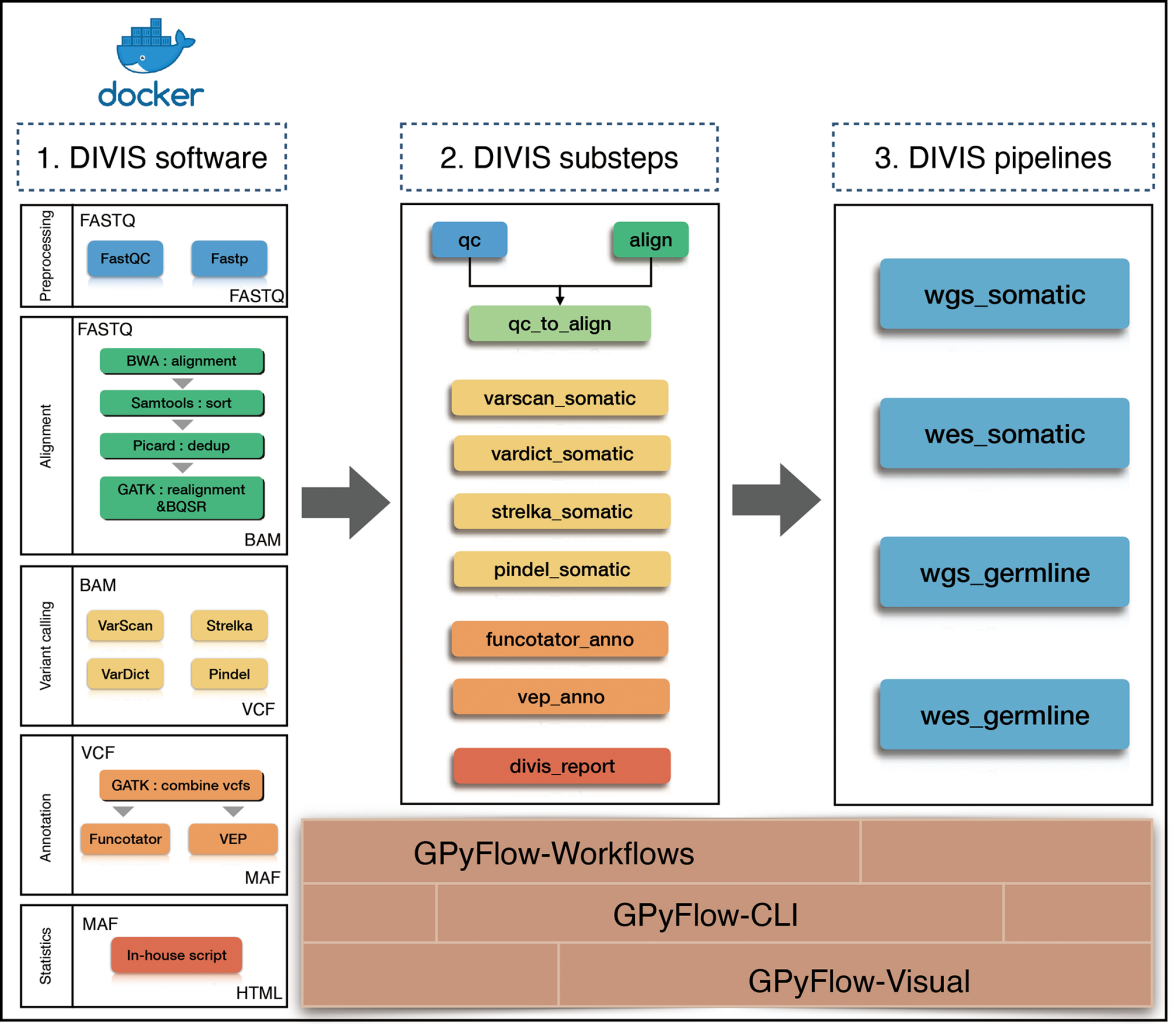
Flowchart of DIVIS pipeline. All steps are managed by GPyFlow and DIVIS runs in two modes: compiled installer and Docker.

To simplify cancer genome sequencing analysis, facilitate workflow extension, and provide accurate mutation results, we presented GPyFlow and DIVIS (variant Detection, Interpretation, Visualization, and Infrastructure), an easy-to-use, extensible, and customizable cancer genome sequencing analysis platform. Here, we introduced the platform’s graphical customization ability, which enables automatic supervision of task scheduling and rapid identification of mutations from WGS, WES, and gene-panel sequencing data. DIVIS allows the identification of reliable and putative somatic SNVs and indels in tumor-normal paired samples or tumor-only samples, and DIVIS also performs germline single nucleotide polymorphism and indel calling in normal samples, which addresses the problems most frequently encountered in the calculations of CDGs and CSGs.DIVIS includes two functional modules, the *pipeline* and *substep* modules (**Figure 1**). Whereas *pipeline* modules integrate a comprehensive and complete workflow from raw sequences to annotate and classify mutation lists, *substep* modules conduct merely single or partial functions during the entire workflow, such as quality control, alignment, and refinement, to detect mutations by using a specific caller or annotation. Users can select *pipeline* or *substep* functions according to application requirements. Both *pipeline* and *substep* modules are implemented using a single command line and record all the procedures and parameters in log files that can be checked anytime.

Users with background knowledge can also launch personalized analyses by adapting the parameters to satisfy their specific research requirements. Moreover, certain parts can also be extended to launch a new *pipeline* or *substep* module. Lastly, for non-expert users, we also presented DIVIS as a Docker (28) image, which eliminates the necessity of any installation and configuration.

## Materials and Equipment

### Test Data and Materials

Six pairs of tumor-normal biological samples with ground truth and standard baseline filtering (variant allele frequency > 0.01, depth of both tumor and normal data were at least 500×) were recruited. The test datasets were provided by the National Center for Clinical Laboratories in China in 2017, 2019, and 2020. The samples were sequenced using an Illumina Hiseq 2500 platform with a target region of 1.76 million bases and an average sequencing depth of 2700×.

### Test Equipment

DIVIS requires a Unix-like operating system. The validation and performance test were conducted on a computer cluster with an Intel Xeon e5-2680v3 processor (2.5 GHz, 12 cores) and Linux machine running CentOS 6.4 with Intel(R) Xeon(R) CPU E5-2680 v2 @2.80GHz.

## Methods

### Implementation

**Figure 2:**
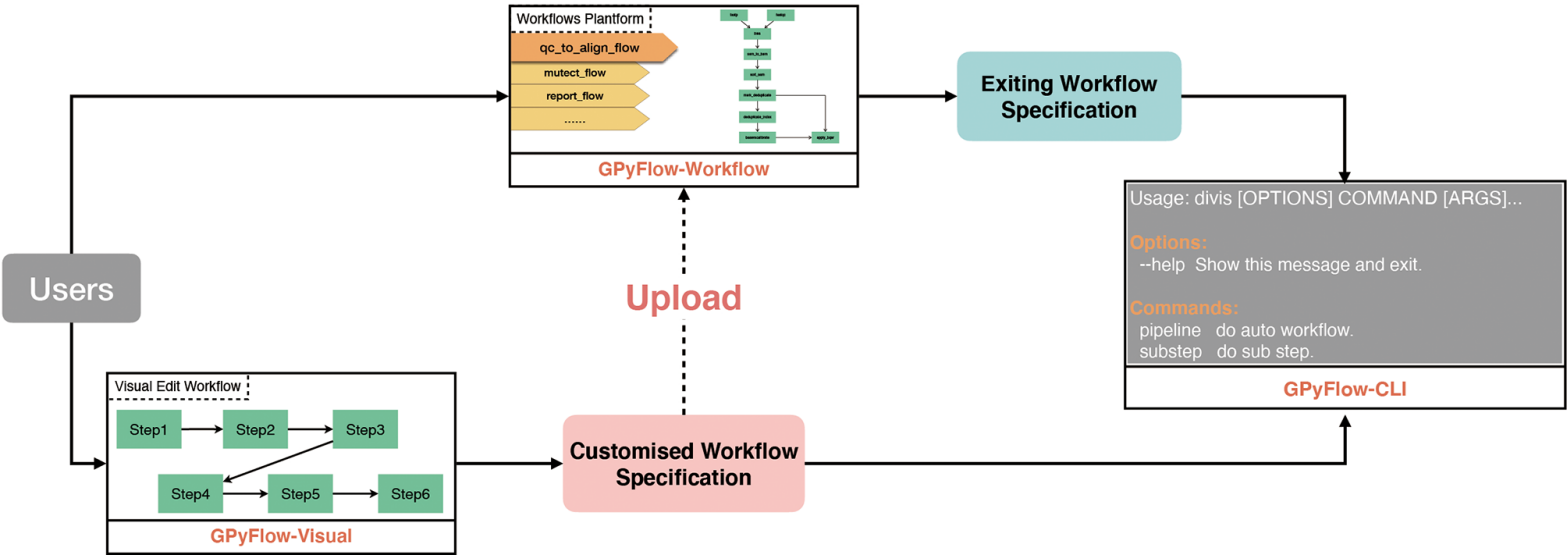
GPyFlow framework.

DIVIS is based on GPyFlow (http://niulab.scgrid.cn/GPyFlow/), which is mainly composed of three discrete modules (**Figure 2**): GPyFlow-Visual, GPyFlow-CLI (GPyFlow-Command-Line Interface), and GPyFlow-Workflows.

**Figure 3:**
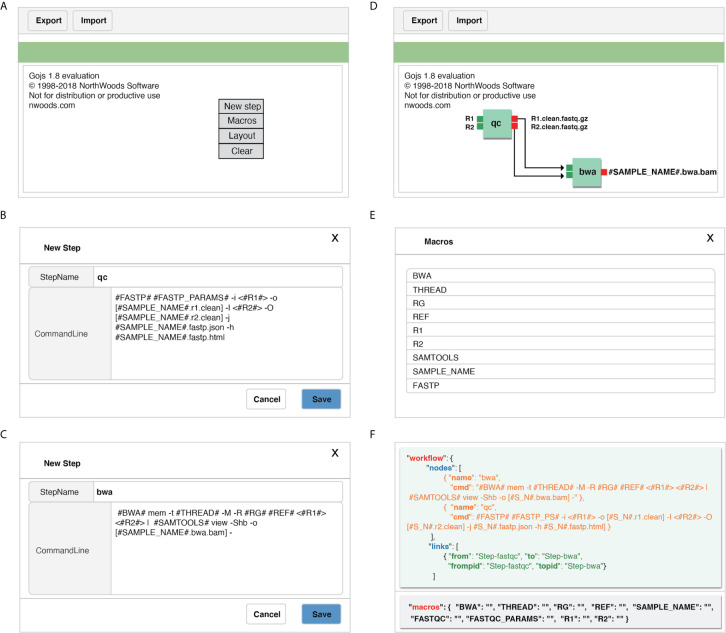
Detailed operations for creating a new step by using GPyFlow-Visual. **(A)** GPyFlow-Visual web portal. **(B)** Edited a new step named “qc”. **(C)** Edited a new step named “bwa”. **(D)** Link step “qc” to “bwa” to make a flow. **(E)** View macros in the flow. **(F)** JSON file of the flow.

GPyFlow-Visual (**Figure 3** **)**, implemented by GoJS (https://gojs.net/latest/index.html) library, provides an interactive graphical operation interface for quickly generating workflow specification (including the name of each step, command lines, and custom macros) by dragging, clicking, and connecting, and this requires no configuration or DSL knowledge. When a command line is typed in the editor, GPyFlow-Visual parses the input marked by “<>,” the output labeled by “[],” and the macro labeled by “##,” and then the background drawing program displays the commands and links on the interface by using rectangles and lines. The input of a step is shown on the left of the rectangles and the output on the right. The detailed operations for creating a new step are as follows:

1. Right-click anywhere on the editing region (**Figure 3A**) to open the operation panel. Click on the “*New Steps*” button to create a new step.
2. Type the desired *StepName*, consisting only of lowercase letters, numbers, and underscores. Use “<>“ in the edit box to indicate the inputs of the step, and use “[]” to display the outputs. The “*#MACRO_NAME#*” field is used to define a macro that can only consist of uppercase letters, numbers, and underscores. This common abstracted information is convenient for workflow reuse (**Figures 3B, C** **)**.
3. After editing, the modules of each step are drawn on the web page, and the macros are extracted. According to the execution order and data transform direction, modules are connected by dragging a line between ports to generate a complete workflow (**Figure 3D**).
4. Click on the “*Macro*” button on the operation panel, and all macros of the workflow can also be viewed (**Figure 3E**).

To facilitate the sharing of workflows, we provide “*Export*” and “*Import*” functions (**Figure 3A**). By using the “*Export*” button, one can save the edited workflow to a JSON file (**Figure 3F**) and submit it to GPyFlow-CLI for execution. One can also import a JSON file to redraw the workflow by using the “*Import*” button. When the “*Export*” function is triggered, GPyFlow-Visual automatically connects and replaces the downstream module’s input with the output of the connected upstream module; subsequently, the newly edited workflow is written to a JSON file. The exported JSON file consists of two fields (**Figure 3F**): The “*macro*” field contains all the macros defined in the workflow that act as temporary placeholders and are initialized genuine input values during execution; the other field, “*workflow*,” contains the name and command lines of each step to be performed. Moreover, the “*from*” statements are for the upstream modules, the “*to*” statements are for the downstream modules, and “*frompid*” and “*topid*” are for the upstream and downstream input ports, respectively.

**Figure 4:**
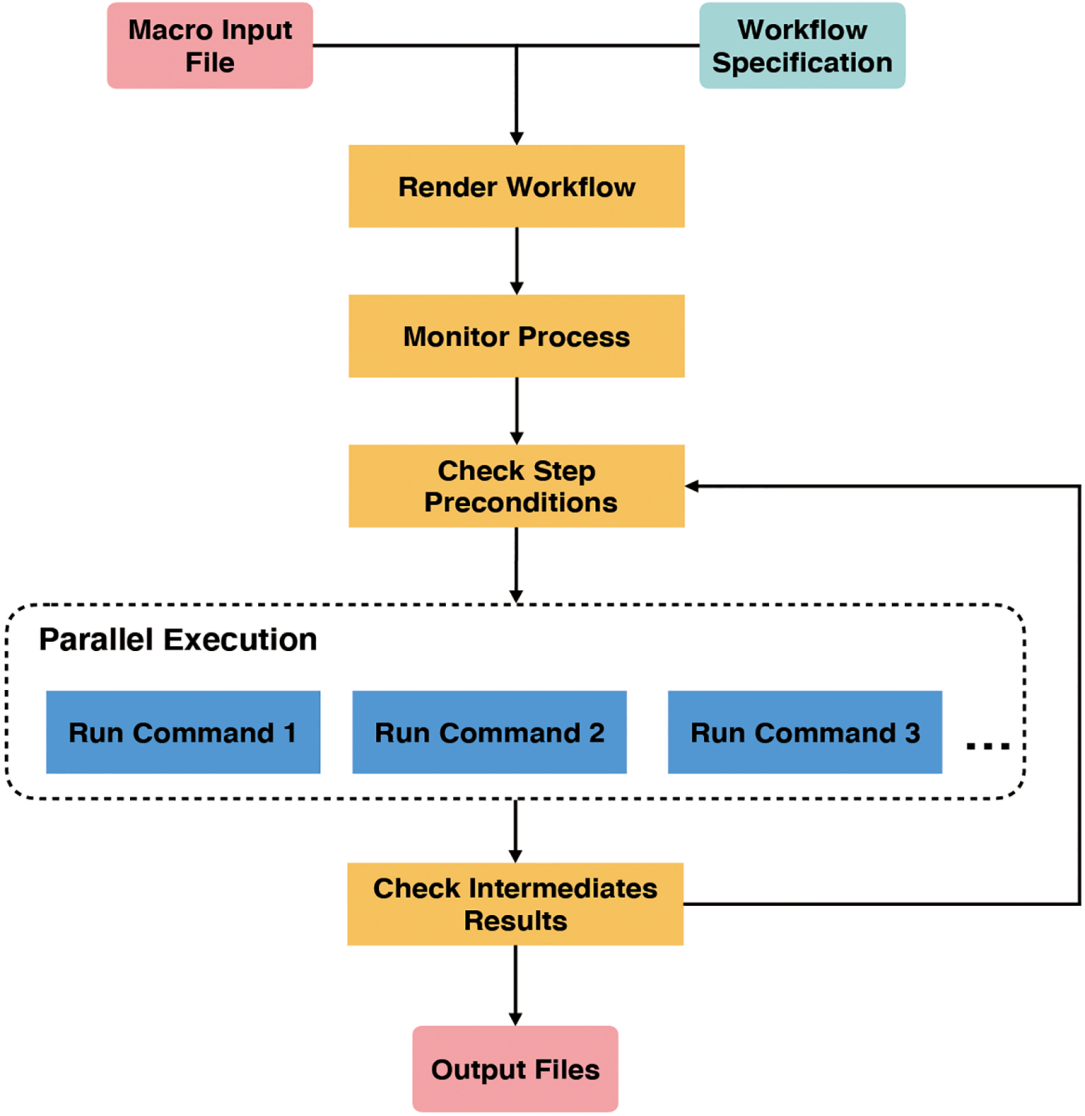
Internal mechanism of GPyFlow-CLI.

GPyFlow-CLI, the core automation component of GPyFlow, schedules and executes command lines in the exported JSON file based on directed acyclic graphs. GPyFlow-CLI is implemented in Python3, and the mechanism is shown in **Figure 4**.

GPyFlow-CLI combines the “*Macro Input File*” with “*Workflow Specification*” to render a workflow that needs to be run. Each workflow contains several interdependent steps. Next, “*Monitor Process*” first runs the steps without pre-dependencies, then updates the interdependency when the steps being run are completed, and, lastly, selects to run the next set of steps without pre-dependencies until all steps have been processed.

In the output directory of GPyFlow-CLI, the following files are mainly used as the auxiliary information of GPyFlow-CLI:

1) ‘[*output_dir*].command.log’ records all executed command lines;
2) ‘[*output_dir*].ok.log’ records successful execution of GPyFlow-CLI, mainly during backtracking when GPyFlow-CLI is not running properly;
3) ‘[*output_dir*].out’ redirects the standard output; and
4) ‘[*output_dir*].err’ redirects the standard error.

GPyFlow-Workflows, implemented using Python-Flask, is a platform for sharing workflows. The platform supports full-text workflow search. Users can not only upload their workflows but also search and download the workflows deposited at GPyFlow. Users can also alter their software parameters or adjust the parameters through a macro interface that can be used and modified by others.

### Substep and Pipeline

We have extensively investigated and tested the bioinformatics tools available for cancer genome sequencing (4). To ensure accurate results and a high running speed, the default software at each stage was ultimately determined.

In the preprocessing stage, fastp (29) and FastQC (30) were used for data preprocessing and quality control (QC), respectively. FastQC summarizes the sequenced GC content, sequencing quality, and several other potential anomalies that are collected and displayed in the final report. BWA (31) MEM was used to align clean reads to the human reference genome (default version: UCSC hg19), and then Picard was applied to sort the aligned reads in coordinate and remove duplicated reads. Next, GATK3.0 and GATK4.11 were used to refine and optimize the mapping by performing local realignment and base quality calibration. For somatic mutations, Strelka2 (v2.9.10) (32), VarDict (v1.8.2) (33), Pindel (v0.2.5b9) (11), and VarScan 2 (v2.3.9) (16) were used to detect SNVs and indels, with the first three tools being used for indels and all four for SNVs. For germline variants, we combined results from Strelka2 (v2.3.9) (32), VarDict (v1.8.2) (33), and GATK HaplotypeCaller (34). Here, we set the local realignment parameters as false (such as –k 0 in VarDict) if the callers perform this function by default, because the function is completed in the alignment step.

The preliminary mutation lists of each caller were processed using in-house scripts; the processing included variant tagging, false-positive filtering, etc. Although we assigned an integer to represent a caller, it cannot reflect the priority of algorithms. By default, the SNVs and indels supported by at least two software tools were selected as candidates.

Somatic mutations were functionally annotated using Funcotator (FUNCtional annOTATOR) (35). Concurrently, germline variants were annotated using Ensembl Variant Effect Predictor (36) and then transformed to the mutation annotation format (MAF). The MAF file, a tab-delimited text file, was proposed and developed by The Cancer Genome Atlas to aggregate mutation information from the VCF files of cohort-level projects. The MAF file is the standard input for downstream advanced analysis tools, such as HotSpot3D (37) for detecting driver genes and MutSigCV (38) for identifying significant mutated genes. Moreover, various tools for processing MAF files have been developed; for example, maftools (39) is a tool specially designed for summarizing, analyzing, and visualizing MAF files. Therefore, DIVIS is compatible with downstream interpretations without format conversion.

Mutated loci featuring a population frequency of >1% were filtered out according to public data sets, including 1000 Genomes (40), The Genome Aggregation Database (41), NHLBI GO Exome Sequencing Project (42), and Exome Aggregation Consortium (43). Next, pathogenic and benign loci were selected based on the evaluation of the scoring software, and CDGs and CSGs were selected and written to the final report.

GPyFlow links all analysis steps. DIVIS automatically runs all steps and records the running status in logs to enable resumption of the task from the nearest error point in case of sudden interruption. The equations should be inserted in editable format from the equation editor.

## Results

**Figure 5:**
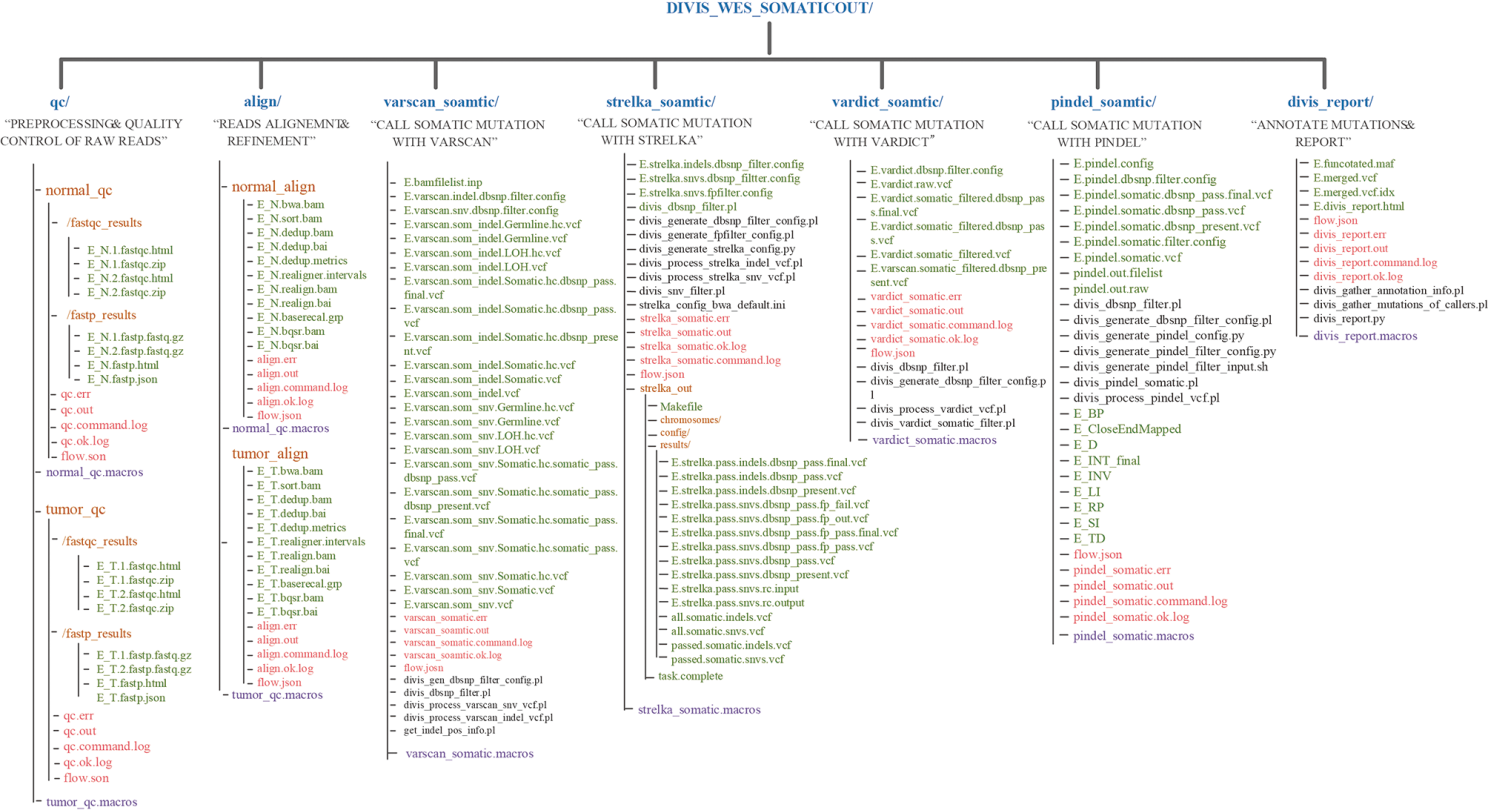
The output directory of DIVIS command “wes_somatic.” In the user specified output directory, “DIVIS_WES_SOMATICOUT,” DIVIS creates independent directories for each “substep,” such as qc/, align/, varscan_ somatic/, etc. The output files related to data analysis are marked in green font. Most of the output files of DIVIS are named incrementally, which makes the file name self-evident. Therefore, users can intuitively judge the specific logical relationship of output files. The purple font marks the macro information of the DIVIS command and the red font marks the auxiliary information of GPyFlow_CLI, including “*.command.log,” “*. ok.log,” “*.out,” and “*. err.”.

DIVIS provided 22 commands **(** **Table 2** **)** in two functional modules and achieved 100% accuracy and recall rate on the test datasets, which indicated outstanding detection ability in cancer genome sequence analysis. The pipeline workflow is divided into four main steps: quality control and preprocessing of FASTQ files, mapping reads to the human reference genome, identification of somatic mutations or germline variants, and filtering and annotation. Each step is encapsulated into the relevant *substep* modules. The concrete scripts are automatically generated and visible, which enable users to adjust parameters to satisfy their specific requirements **(** **Figure 5** **)**.

**Table 2:** Descriptions of DIVIS modules “Pipeline” and “Substep”.

	Modules	Descriptions	Commands
pipeline	Somatic Mutation Calling	Call somatic mutations from Tumor-Normal paired FASTQ files, generate intermediate VCF files, then convert mutations to MAF file for WES, WGS and gene-panel	wes_somatic wgs_somatic
	Germline Variants Calling	Call germline variants from Normal	wgs_germline wes_germline
		samples and generate VCF files	
Substep	Preprocessing	Preprocessing raw FASTQ files to clean	qc
		FASTQ files and generate QC reports	
	Alignment and refinement	Alignment from FASTQ to BAM: BWA	
		alignment, sort, remove duplications, local realignment and BQSR	align
	Preprocessing-Alignment	Preprocessing+Alignment	qc_to_align
			varscan_somatic
			strelka_somatic
	Mutations or Variants calling	Calling mutations or variants from BAM	pindel_somatic vardict_somaitc mutect1_somatic gatk4_haplotypecall
		files and then generate VCF files	er_germline
	Annotation	Convert mutations or variants from VCF files to MAF files	oncotator funcotator vep

DIVIS provides a statistics report of all the steps and ultimate mutations; the report contains three parts: The first part shows the command lines for data processing and generation. The second part shows the quality-control metrics and statistics of the sequencing, mapping, and calling stages and helps users obtain the necessary information from a global perspective, which also provides evidence for fine-tuning parameters. In the third part, DIVIS provides the mutation landscape, which encompasses the proportion of mutation types, transitions/transversions ratios, tumor mutation burdens, CDGs, CSGs, etc. This HTML-format report featuring interactive figures and tables (https://github.com/niu-lab/DIVIS/blob/master/divis_report_demo.html) makes it convenient for colleagues to share, collaborate on, and rapidly reproduce their work.

We tested DIVIS on a single node of supercomputer “ERA” which equipped with Intel Xeon E5-2680V3 (2.5 GHz, 12 cores) and with single node CPU computing power of 0.96 Tflops. The DIVIS commands were submitted to (bsub) to CPU queue (**Table 3**). Command “wgs_somatic” costed 3 days, 8 h, and 33 min. The corresponding general processing time is 5 days and 12 h. DIVIS also saved more than 3 h in WES data with the normal process, the time of divis is mainly saved in the time of each mutation identification software. Improvement of efficiency of DIVIS is mainly due to the parallelization of independent mutation calling process.

**Table 3:** Execution time statistics of DIVIS.

Environment	Command	Sequencing	Depth	Data Size	Execution Time	General Time
ERA(c_bniu)	wgs_somatic	WGS	T: 60× N: 29×	T: 257G N: 129G	3d8h33m	5d12h0m
ERA(c_bniu)	wes_somatic	WES	T: 290× N: 105×	T: 12G N: 4.7G	11h31m	14h55m

## Discussion

As a data-intensive discipline, cancer genome sequencing frequently requires a series of data-processing steps that demands a considerable amount of bioinformatics knowledge. We presented GPyFlow, a tool mainly composed of three modules, GPyFlow-Visual, GPyFlow-CLI, and GPyFlow-Workflows, to define, edit, and automatically execute workflows. Through its lightweight design and support of visual editing and free sharing, GPyFlow will appreciably enhance the ability of users to test and reuse distinct workflows. Based on GPyFlow, we built DIVIS, an integrated software providing *pipeline* and *substep* modules for completing common tasks in cancer genome sequencing, such as identifying CDGs from somatic mutations and CSGs from germline variants. DIVIS performs the required alignment and calling and ultimately generates a MAF mutation list, which is highly convenient for downstream advanced mining and interpretation. Moreover, the generated statistical reports summarize quality indicators of sequencing, alignment, and calling, various callers’ intersection, tumor mutation load, etc.

Although in this work we validate the performance of DIVIS with gene-panel sequencing samples, more types of sequencing datasets are necessary to comprehensively demonstrate the advantages of DIVIS. Moreover, DIVIS currently does not cover certain types of variants. Next, DIVIS will be updated according to an iterative development model to include CNVs and SVs. DIVIS will also be designed to attempt advanced interpretation of cancer cohort analysis, mainly including mutational signatures (44), significantly mutated genes (45), mutation hotspots in protein 3D structures (37), biological pathways and biomarkers (46, 47), etc. DIVIS will cover as many mutation signals as possible in a one-stop manner, and provide a viable solution for the development and use of processes for analyzing biological information.

## Data Availability Statement

The data analyzed in this study is subject to the following licenses/restrictions: The datasets for this study were provided by the National Center for Clinical Laboratories in China and will be shared at reasonable request to the corresponding author. Requests to access these datasets should be directed to niubf@cnic.cn.

## Author Contributions

BN contributed to the conception of the study and supervised the research. XiaoH and YZ participated in the design of the study and wrote the manuscript. DY performed the experiment with constructive discussions. XinH participated in the manuscript preparation, specifically visualization. XD, SL, XW and JH conducted the investigation and manuscript editing. All authors contributed to the article and approved the submitted version.

## Funding

This work is supported by the Strategic Priority Research Program of the Chinese Academy of Sciences [grant number XDB38040100]; the National Natural Science Foundation of China [grant number 31771466]; the Cancer Genome Atlas of China (CGAC) project (YCZYPT[2018]06) from the National Human Genetic Resources Sharing Service Platform (2005DKA21300).

## Conflict of Interest

Authors XD, SL, XW was employed by company ChosenMed Technology (Beijing) Co., Ltd.

The remaining authors declare that the research was conducted in the absence of any commercial or financial relationships that could be construed as a potential conflict of interest.
